# Non-synonymous ERG11 mutations in *M. restricta* and *M. arunalokei*: impact on azole susceptibility

**DOI:** 10.1128/spectrum.00007-25

**Published:** 2025-05-15

**Authors:** Cheryl Leong, Wisely Chua, Cheng-Shoong Chong, Shi Mun Lee, Sebastian Maurer-Stroh, Won Hee Jung, Thomas L. Dawson

**Affiliations:** 1 A*STAR Skin Research Labs (A*SRL), Agency for Science, Technology and Research (A*STAR) & Skin Research Institute of Singapore (SRIS) 726618 , Singapore, Singapore; 2 Bioinformatics Institute, Agency for Science, Technology and Research (A*STAR) 54759, Singapore, Singapore; 3 Department of Biological Sciences and Yong Loo Lin School of Medicine, National University of Singapore (NUS) 145755 , Singapore, Singapore; 4 Department of Systems Biotechnology, Chung-Ang University, Seoul, South Korea; 5 Center for Cell Death, Injury & Regeneration, Department of Drug Discovery and Biomedical Sciences, Medical University of South Carolina 2345, Charleston, South Carolina, USA; Universidade de Sao Paulo, Ribeirao Preto, Sao Paulo, Brazil

**Keywords:** antifungal, resistance, azoles, *Malassezia*, ERG11

## Abstract

*Malassezia* are commensal lipid-dependent yeasts and opportunistic pathogens that cause superficial mycoses and systemic infection. Azole antifungals target cell wall ergosterol synthesis and are the first line of antifungal treatment. ERG11 gene mutations and overexpression are major mechanisms conferring azole resistance and resulting in antifungal therapy failure. *Malassezia restricta* is found ubiquitously on healthy and diseased skin, with azole-resistant isolates described. *Malassezia arunalokei* is a relatively new, closely related common skin species. Ketoconazole and itraconazole were the most effective at inhibiting both species. Isolates of *M. restricta* and *M. arunalokei* from healthy skin of Singapore subjects were cultured, evaluated, and generally susceptible to common over-the-counter azoles, including clotrimazole, except for select less-susceptible strains. Some less-susceptible strains have novel or reported non-synonymous mutations in the ERG11 gene, such as R88C. The QK178RQ ERG11 sequence variation was observed to be associated with differences in *M. restricta* and *M. arunalokei* as independent species. In the absence of identified ERG11 mutations, strains with elevated MICs were observed to have elevated ERG11 expression and drug efflux pump expression/activity. We conclude that antifungal susceptibility is determined by a combination of intrinsic (e.g., mutations, gene expression, efflux pump activity) and extrinsic (e.g., skin condition, prior antifungal exposure) factors and that the skin microbiome serves as a reference for the emergence of new mutations and strain phenotypes.

**IMPORTANCE:**

*Malassezia* over colonization is associated with conditions such as dandruff and seborrheic dermatitis, which give rise to unpleasant itching and swelling on the skin. Azole antifungals such as ketoconazole, clotrimazole, and miconazole are the primary treatments of choice available as over-the-counter creams or shampoos. However, the emergence of antifungal resistance leads to a loss of treatment efficacy and persistent fungal infection. To understand the mechanisms underlying antifungal resistance, we profiled the susceptibility profiles of commensal *Malassezia* isolates from the skin and identified novel ERG11 mutations. Our results indicate that antifungal susceptibility is determined by a combination of factors (mutations, efflux pump activity, gene expression, copy number) and suggest that the healthy skin microbiome serves as a reference for the emergence of new mutations and strain phenotypes.

## INTRODUCTION

*Malassezia* comprise a species of lipid-dependent commensal yeasts commonly occupying skin. Eighteen species are presently described, with 10 being mainly anthropophilic*—M. globosa*, *M. restricta*, *M. sympodialis*, *M. furfur*, *M. slooffiae, M. arunalokei, M. japonica, M. yamatoensis, M. obtusa,* and *M. dermatis* (1, 2). *M. restricta* is the most common and abundant *Malassezia* species on human skin as detected by genomic sequencing methods (3). However, it is less easily detected in culture due to its fastidious growth requirements and slow growth (4). Ketoconazole-resistant *M. restricta* strains have been described in dandruff patients associated with tandem gene multiplication and increased drug efflux pump activity (5, 6).

*Malassezia arunalokei* is the newest *Malassezia* species described and has been isolated from the scalp and nasolabial folds of healthy and seborrheic dermatitis patients in India (7). They are postulated to be sibling species, having evolved from a common ancestor by translocation between the pericentromeric and non-centromeric regions of two different chromosomes (1). Both species have nine chromosomes of similar size, with 93.5% nucleotide similarity (3). Since its discovery, *M. arunalokei* has also been found to be common on human skin (3, 7, 8), although little has been described about its antifungal susceptibility and its role in host-microbe interactions.

*Malassezia* over colonization is commonly associated with seborrheic dermatitis, pityriasis versicolor, and other opportunistic fungal infections (9). These conditions are characterized by itch, swelling, and an unpleasant skin rash which may persist and spread onto large areas if left untreated. For superficial infections, azole antifungal drugs such as ketoconazole, clotrimazole, and miconazole are the primary treatments of choice and widely available as over-the-counter creams or shampoos. They are also used prophylactically for extended periods to prevent infection recurrence (10). Immunocompromised individuals are at risk of systemic infection, and outbreaks in neonatal units associated with pathogenic strains of *M. furfur* and *Malassezia pachydermatis* have been reported (11, 12). In systemic infection, azoles such as itraconazole and fluconazole may be used orally or systemically but not without side effects, and these treatments may have limited efficacy depending on the strain susceptibility.

Azole-resistant isolates of *Malassezia* have been characterized in *M. furfur*, *M. restricta*, *M. globosa,* and *M. pachydermatis* (5, 6, 13, 14). Non-synonymous mutations located at hotspots in the ERG11/CYP51A1 (15) gene, which encodes for lanosterol 14 alpha-demethylase that is involved in ergosterol biosynthesis, an essential fungal cell wall component, are the most commonly described mechanism of azole resistance (16). Several of these mutations have also been described for *M. restricta*, *M. furfur,* and *M. globosa* (5, 6, 13). The expression and cellular activity of the pleiotropic drug transporters PDR5 and PDR10 and the ABC transporter mitochondrial 1 (ATM1) have been identified to play an important role in multidrug resistance. Identification of the correct gene loci in *Malassezia* is challenging due to the absence of well-annotated validated genomes for all species and the presence of multiple copies of highly homologous genes. Gene multiplications in ERG4, ERG11, and ATM1 have also been described in *M. restricta* and *M. pachydermatis* (5, 14). Together with the increased expression of drug efflux pumps, these mechanisms contribute to the overall resistance of these strains to currently available azole drug treatments (17).

In this study, we compared the antifungal susceptibility profiles of *M. restricta* isolates obtained from Singapore and Korea using a previously developed antifungal susceptibility assay which caters to the lipid requirements of *Malassezia* (18). We hypothesize that antifungal drug susceptibility of species may vary with strain prevalence and geographical location. We also describe for the first time the antifungal susceptibility of five *M. arunalokei* strains, four of which are local isolates collected in Singapore.

## MATERIALS AND METHODS

### *Malassezia* strains and culture conditions

The *Malassezia* reference strains *M. restricta* (CBS 7877) and *M. arunalokei* (CBS 13387) were obtained from the Westerdijk Fungal Diversity Institute. No primary culture isolation was performed for this study. The *M. restricta* clinical isolates KCTC 27529, 27550, 27524, 27527, 27539, and 27540 were obtained from Korean patients with severe dandruff as previously described (19). The remaining *M. restricta* and *M. arunalokei* strains were obtained from the skin of healthy subjects in Singapore as previously described (20). Cultures were maintained in modified mDixon broth comprising (per 1 L) 20 g desiccated Oxbile (B3883, Sigma Aldrich, Singapore), 6 g peptone (BD 211677, Becton Dickinson, Singapore), 36 g malt extract (70167, Sigma-Aldrich, Singapore), 2 mL oleic acid (27728, Sigma Aldrich, Singapore), 10 mL Tween 40 (P1504, Sigma Aldrich, Singapore), and 4 mL 50% glycerol (BUG1120, 1st Base, Singapore) for 3–5 days at 32°C.

### Broth microdilution for antifungal susceptibility testing

Antifungal susceptibility testing was performed using a broth microdilution method as described by Leong et al. (18). Amphotericin B, terbinafine, clotrimazole, miconazole, itraconazole, fluconazole, voriconazole, and ketoconazole were purchased from Sigma-Aldrich, Singapore. Drug stock dilutions were prepared at a 200× concentration in fresh OptiMAL medium (18), in accordance with CLSI and EUCAST guidelines. Yeast inocula were obtained from fresh cultures of *Malassezia*. A 50 µL yeast inoculum was added to 50 mL of 2× concentrated antifungals to achieve a final cell density of 5 × 10^3^ to 5 × 10^4^ CFU/mL. Another 10 µL of yeast inoculum that had been diluted 10 times was also plated onto a mDixon agar plate and incubated for 4 to 7 days at 35°C for post verification of the CFU inoculum (10 to 100 colonies per plate). Each assay was performed in triplicate plates for a single culture at every individual time point or reading.

### Identification of *M. restricta* PDR5, PDR10, and ATM1 homologs

To identify and validate the relevant gene sequences for ERG11, PDR10, and ATM1 in *M. restricta*, *Saccharomyces cerevisiae* protein sequences were used as a reference. The reference IDs and percentage identification for each protein are shown in Table 1 below. These gene sequences were used for subsequent primer design and gene analysis of the respective genes.

**TABLE 1:** *Malassezia* gene sequences as identified from *S. cerevisiae* protein sequence identity

*S. cerevisiae*	Identity	Reference	ID
ERG11			
*M. furfur*	46.36%	KAI 3624029	ERG11
*M. globosa*	48.27%	XP_001730619.1	Uncharacterized protein MGL_2415
*M. restricta*	47.27%	XP_027485371.1	Sterol 14-demethylase/MRET_3233
ATM1			
*M. furfur*	56.38%	KAI3622948.1	ATM1
*M. globosa*	57.57%	XP_001730093.1	Uncharacterized protein MGL_2475
*M. restricta*	57.59%	XP_027486643	Mitochondrial ABC transporter ATM/MRET 4198
PDR10			
*M. furfur*	36.94%	KAI3624285.1	Hypothetical protein CBS14141_002713
	27.94%	KAI3627486.1	Hypothetical protein CBS14141_001487
*M. restricta*	37.83%	XP_027484064.1	ABC transporter (True PDR10)/MRET_2329
	36.61%	XP_027484740.1	ABC transporter (True PDR5)/MRET_2330
*M. globosa*	37.52%	XP_001732727.1	Uncharacterized protein MGL_0502 (True PDR5)
	38.85%	XP_001732726.1	Uncharacterized protein MGL_0501 (True PDR10)

### Gene identification and analysis

Primers used for the amplification of the relevant gene regions are listed in Table S1. Gene sequences derived from Sanger sequencing were translated into protein sequences using the ExPASy translate tool (https://web.expasy.org/translate/) and analyzed using multiple-sequence alignment (http://multalin.toulouse.inra.fr/multalin/multalin.html). Phylogenetic tree analysis was performed after multiple-sequence alignment of all internal transcribed spacer (ITS) sequences using Clustal Omega (21).

PCR reactions were performed in a 10 µL reaction volume comprising 1 µL of 10× PCR reaction buffer without magnesium chloride (Invitrogen, Singapore), 0.05 µL Platinum Taq DNA polymerase (Thermo Fisher, Singapore), 0.4 µL of 2 mM magnesium chloride (Promega, Singapore), 0.2 µL of 0.2 mM dNTP (Promega, Singapore), and 7.6 µL of nuclease-free water on a ProFlex PCR systems thermocycler (Applied Biosystems, Singapore). The thermal cycle was programmed for 90 s at 95°C for initial denaturation, followed by 35 cycles of 30 s at 95°C for denaturation, 1 min for annealing, 2 min at 72°C for extension, and 7 min at 72°C for the final extension. PCR products were examined by electrophoresis at 120 V for 30 min in a 1% (wt/vol) agarose gel in 1× Tris-acetate-EDTA (TAE) buffer and visualized using Floro+ Red nucleic acid stain (1st Base, Singapore). Sanger sequencing was performed using the BigDye Terminator v.3.1 cycle sequencing kit (Thermo Fisher, Singapore) using an ABI PRISM 3730xl Genetic Analyzer (Applied Biosystems, USA) with KB Basecaller as per manufacturer’s instructions.

For quantification of gene expression, RT-qPCR was performed using the GoTaq 1-Step RT-qPCR system (Promega, Singapore) on RNA extracted from the respective strains with gene-specific primers using *Malassezia* actin *ACT1* as the housekeeping gene. Relative gene expression was analyzed using the Qqene module as described by Muller et al. (22). For gene copy number quantification, genomic DNA was used as template for gene-specific qPCR quantitation (Luna Universal qPCR Master Mix, New England Biolabs, Singapore) using *Malassezia* actin *ACT1* as a single copy number reference for normalization. qPCRs were run on a QuantStudio 6 Flex real-time PCR system (Applied Biosystems, Singapore) using the cycling conditions as per the recommended Master Mix kits.

### ERG11 homology protein modeling

Models of the wild-type and mutant ERG11 proteins were generated by aligning *M. restricta* and *M. arunalokei* ERG11 sequences produced in this study with experimentally determined wild-type ERG11 structures from *S. cerevisiae* (PDB ID: 4WMZ) and *Candida albicans* (PDB ID: 5V5Z) using Clustal Omega (21). The alignment file was used to build protein homology models in SWISS-MODEL (23–25) using the “Target Template” input methods, and the best model(s) based on Global Model Quality Estimate score was chosen for analysis. All images were produced using CHIMERA (26).

### Rhodamine 6G efflux assay

All strains of *M. restricta* and *M. arunalokei* were grown to log phase and normalized to an OD_600_ = 0.1. Cells were washed twice in phosphate buffered saline (PBS) followed by loading with 10 mM of rhodamine 6G dye (R4127, Sigma-Aldrich, Singapore) for 30 min at 32°C. After loading, cells were washed twice in cold PBS and maintained at 32°C. Cell suspension was collected at intervals of 0, 15, and 30 min and spun down to collect the supernatant. Triplicate readings were taken per strain, and rhodamine 6G fluorescence was measured at excitation/emission 425/520 nm (Varioskan LUX, Thermo Fisher Scientific). Values were plotted as a fold change over the 0 min reading.

## RESULTS

### Ketoconazole and itraconazole are most effective for inhibiting all strains of *Malassezia*

**Fig 1:**
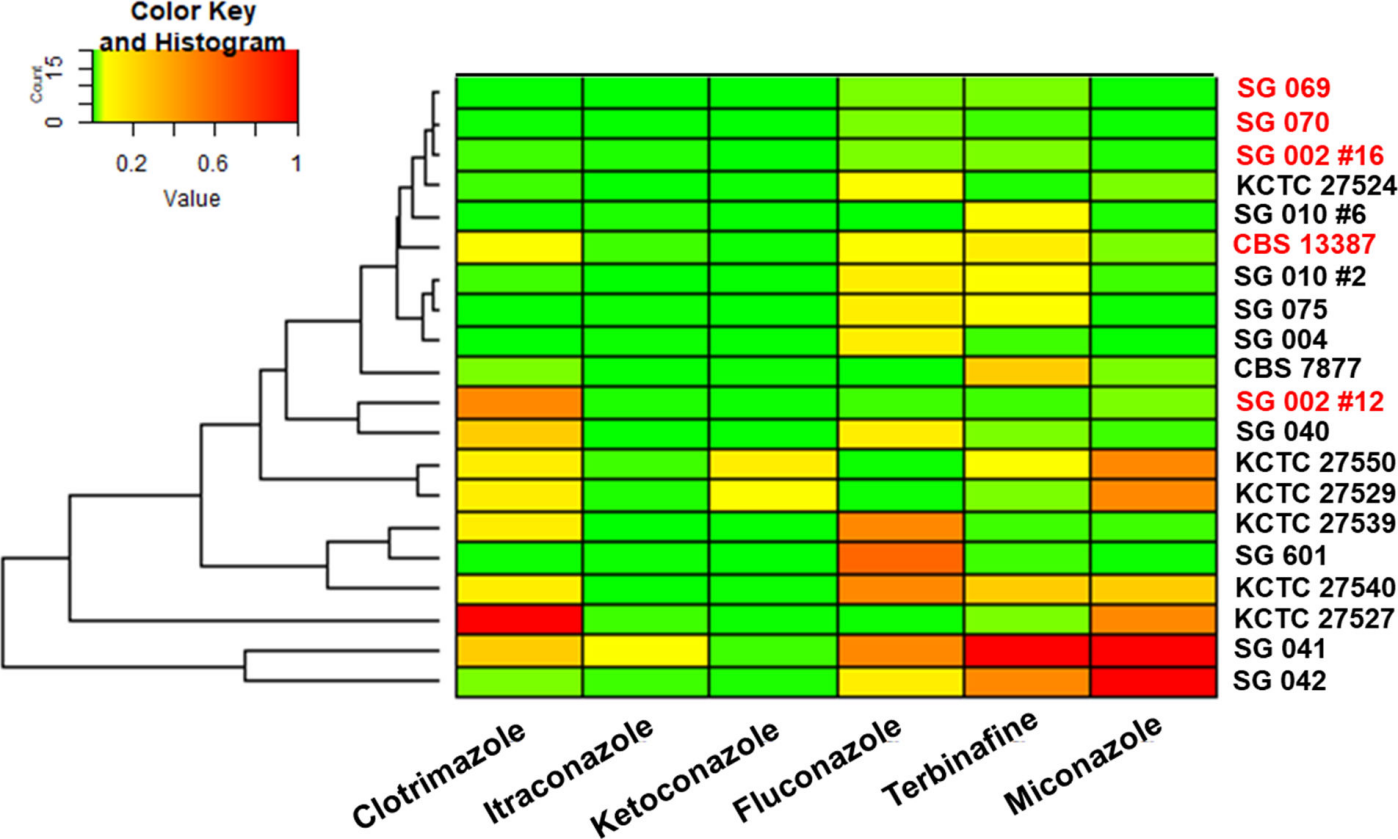
Heatmap of the MIC values of six antifungals. MIC values were normalized from 0 to 1 with 1 being the highest MIC value (red) for each individual compound. Strains in red are *M. arunalokei* and strains in black are *M. restricta*.

Six commonly used antifungals were tested—clotrimazole, miconazole, ketoconazole, terbinafine, itraconazole, and fluconazole. Ketoconazole and itraconazole had the lowest MICs for all strains of *Malassezia* tested (Fig. 1; Table 2). *M. restricta* strains KCTC 27550 and 27527 were the only two strains with elevated MICs for ketoconazole. This is consistent with previous reports that they are ketoconazole resistant (5). Elevated MICs were observed for the isolates KCTC 27550, 27527, 27529, 27539, and 27540 (Fig. 1; Table 2). This may be associated with their disease background, where the strains were isolated from Korean patients with severe dandruff. Two isolates from healthy subjects in Singapore, strains SG 041 and SG 042, were observed to have elevated MICs for fluconazole, terbinafine, and miconazole, which suggest they may have intrinsic mechanisms of antifungal resistance.

**TABLE 2:** List of *Malassezia* strains tested

Strain ID	Source	Species	MIC range (μg/mL)/(mean MIC)					
			Itraconazole	Ketoconazole	Clotrimazole	Fluconazole	Terbinafine	Miconazole
SG 004	Singapore, healthy	*M. restricta*	0.03–0.06	<0.008	0.015	1–2	0.125–0.5	0.015–0.03
SG 010 #2	Singapore, healthy	*M. restricta*	0.008–0.015	0.03	0.125–0.25	2–8	0.5–1	0.125–0.25
SG 010 #6	Singapore, healthy	*M. restricta*	0.06–0.125	0.008–0.06	0.03–0.25	0.125–2	0.06–2	0.06–0.125
SG 040	Singapore, healthy	*M. restricta*	0.015–0.03	0.03	2–4	32	0.25–0.5	0.25
SG 041	Singapore, healthy	*M. restricta*	0.5–1	0.25	2–4	4–8	8–16	64–128
SG 042	Singapore, healthy	*M. restricta*	0.03–025	0.0625–0.125	0.5–1	32	2–8	16–32
SG 075	Singapore, healthy	*M. restricta*	0.03–0.06	≤0.008	0.03	0.5–2	1–4	0.03–0.06
SG 601	Singapore, healthy	*M. restricta*	0.03–0.25	0.008–0.06	0.03–0.06	0.5–2	0.25–1	0.03–0.25
SG 002 #12	Singapore, healthy	*M. arunalokei*	0.06–0.125	0.06	8	4	0.25	0.5
SG 002 #16	Singapore, healthy	*M. arunalokei*	0.125–0.25	0.015	0.25	4–8	0.5	0.06–0.125
SG 069	Singapore, healthy	*M. arunalokei*	<0.008–0.015	<0.008	0.03	8	0.25–0.5	0.06
SG 070	Singapore, healthy	*M. arunalokei*	0.015–0.03	<0.008	0.03–0.06	8	0.06–0.25	0.06
KCTC 27524	Korea, dandruff	*M. restricta*	4–8	0.06	0.25–0.5	8–16	0.125	0.5
KCTC 27527	Korea, dandruff	*M. restricta*	0.125–0.25	0.03–0.06	16	0.5–1	0.25–0.5	4–8
KCTC 27529	Korea, dandruff	*M. restricta*	0.06–0.125	0.05–0.25	2.0	0.5–1	0.25–0.5	8–16
KCTC 27539	Korea, dandruff	*M. restricta*	0.015	0.03	1–2	8	0.25	0.25
KCTC 27540	Korea, dandruff	*M. restricta*	0.015–0.03	0.03–0.06	1–2	4–8	2–4	2–4
KCTC 27550	Korea, dandruff	*M. restricta*	0.125–0.25	1–2	16	32–64	1	8–16
CBS 7877	Type strain	*M. restricta*	0.015–0.03	0.03–0.06	0.5	0.25–0.5	4	0.5
CBS 13387	Type strain	*M. arunalokei*	0.125–0.25	0.03–0.125	0.5–1	8–16	1–4	0.25–0.5

### The non-synonymous mutation QK-RQ allows *M. arunalokei* species discrimination from *M. restricta*

A total of 12 primary *Malassezia* isolates were originally identified as *M. restricta* from Singapore in 2019 (20). They were screened for antifungal susceptibility using the broth microdilution method alongside six clinical isolates of *M. restricta* from Korea (5). With the inclusion of the *M. arunalokei* genome into the NCBI database in 2023, four (002 #12, 002 #16, 069, 070) of these 12 strains were re-classified as likely *M. arunalokei* based on comparison between ITS and ERG11 multiple-sequence alignments (Fig. S1). As such, the *M. arunalokei* reference strain, CBS 13387, was also included.

**Fig 2:**
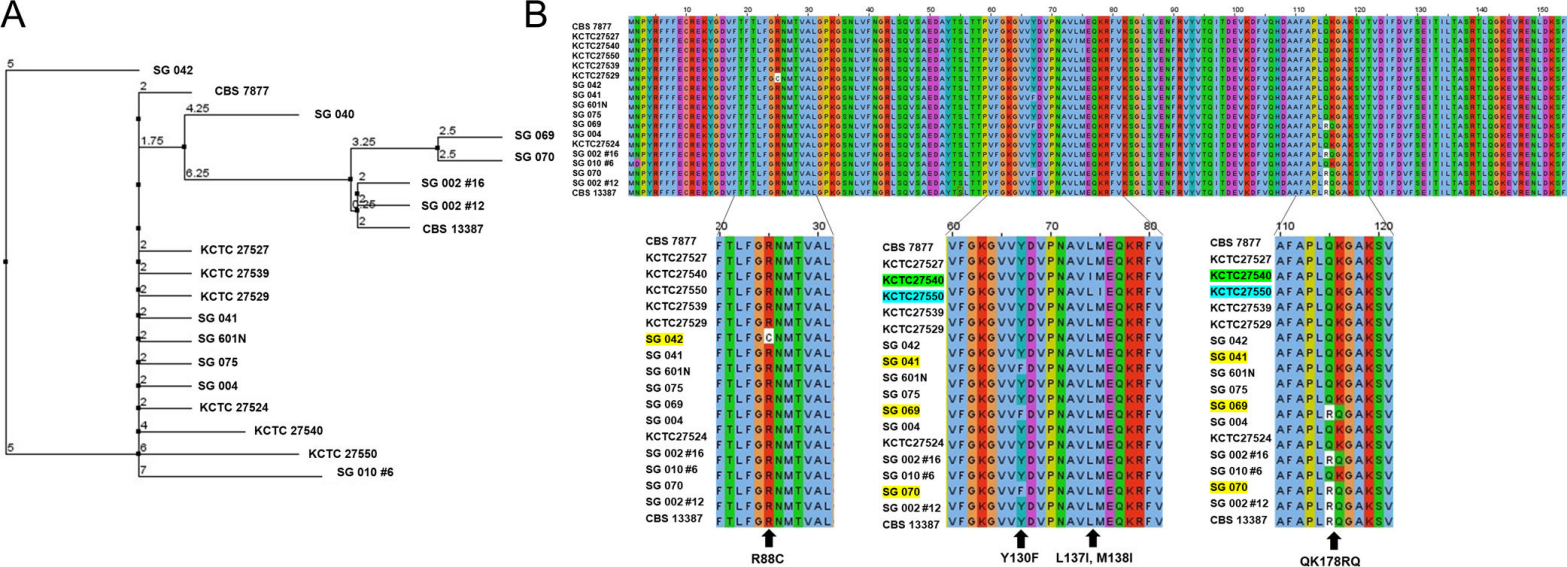
(**A**) Phylogenetic tree based on ERG11 amino acid sequences and (**B**) non-synonymous mutations in ERG11 homolog of *M. restricta* and *M. arunalokei* strains.

Of the original ERG11 Sanger sequencing results from all 12 isolates, we identified four strains with a distinct QK178RQ mutation. Phylogenetic tree analysis of the ERG11 sequence shows that these strains, together with *M. arunalokei* reference strain, CBS 13387, form a distinct cluster (Fig. 2A). With the reclassification of the five strains of *M. arunalokei*, it appears that the QK178RQ sequence variation is unique to *M. arunalokei* and may be used to differentiate it from *M. restricta*. While *M. arunalokei* isolates were observed to have higher MIC values for fluconazole compared to *M. restricta*, this non-synonymous mutation cluster was not observed or predicted to be associated with antifungal susceptibility (5, 27, 28).

### The R88C mutant displays elevated MICs for fluconazole, terbinafine, and miconazole

To determine genomic mutations underlying the elevated MICs observed in strains SG 041 and SG 042, we performed targeted amplification and sequencing of the ERG11 gene in all strains. Six non-synonymous mutations were observed in the ERG11 gene spanning amino acid regions 1 to 150 (Fig. 2B; Table 3), four of which have been previously described. The novel mutation, R88C (strain SG 042), was observed to be associated with elevated MICs for fluconazole, terbinafine, and miconazole. All other mutations have been previously reported, except for QK178RQ, which, as discussed above, may be used in species discrimination between *M. arunalokei* and *M. restricta*.

**TABLE 3:** Non-synonymous mutations in ERG11

Mutation	Strain ID	Reported
R88C	SG 042	–^*a*^
Y130F	SG 040, SG 069, SG 070	(5, 6, 20)
L137I	KCTC 27540	(5)
M138I	KCTC 27550	(5)
Q178R	SG 002 #12, SG 002 #16, SG 069, SG 070	(5, 27)
K179Q	SG 002 #12, SG 002 #16, SG 069, SG 070	–

^*a*^ – indicates these mutations have not been previously reported.

To evaluate the impact of this single amino acid change on ERG11 protein structure and function, we performed *in silico* protein homology modeling using the *S. cerevisiae* ERG11 protein as a reference structure. This is based on the premise that the P450 (CYP) family of proteins has been established to be highly homologous, and the sequence identity between *M. restricta* and *S. cerevisiae* falls within acceptable ranges (>25%) established for successful modeling (21).

**Fig 3:**
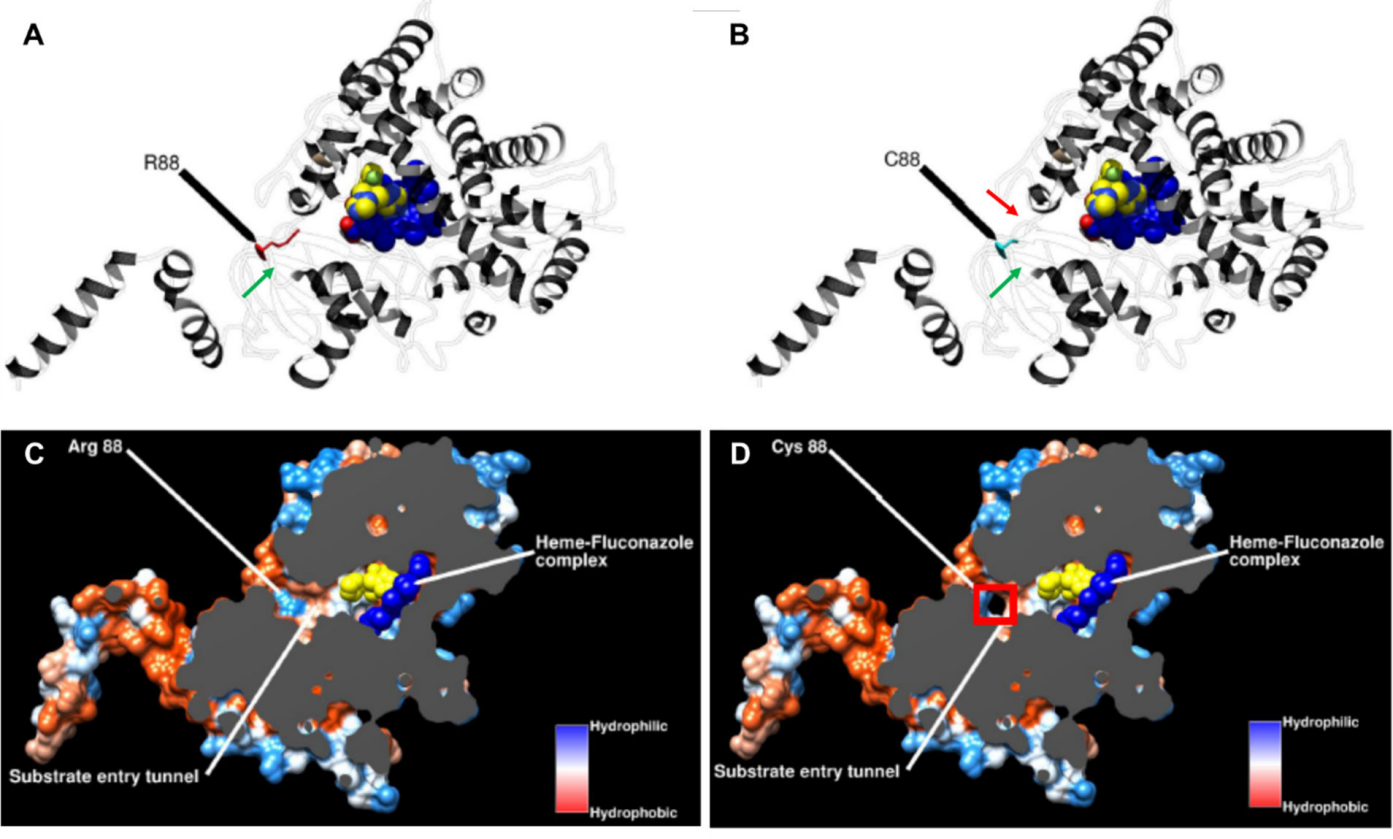
Frontal view of *M. restricta* ERG11 (**A**) wild type and (**B**) R88C mutant. Wild-type R88 is shown in red, while mutant C88 is in cyan. Heme-fluconazole complex is shown in the substrate binding pocket—prosthetic heme group in blue, fluconazole in yellow. Green arrows indicate the main substrate access channel. Red arrow indicates the secondary pore. Secondary structures are transparent for clarity. Image prepared with CHIMERA (26). Surface frontal view of *M. restricta* ERG11 (**C**) wild type and (**D**) R88C mutant. Structure is clipped by plane (gray) to show the substrate entry tunnel. Hydrophobic surfaces in red, hydrophilic surfaces in blue. Heme-fluconazole complex is shown in the substrate binding pocket—prosthetic heme group in blue, fluconazole in yellow. Predicted secondary pore into substrate entry tunnel is boxed in red. Image prepared with CHIMERA (26).

*In silico* protein homology modeling shows that the R88C mutation occurs near the opening of the substrate entry hydrophobic tunnel, which allows for both substrate and inhibitor entry (Fig. 3). This region is highly conserved across Basidomycota (29). Cytochrome P450 proteins tend to have highly conserved active sites that are buried deep within the protein. Thus, the substrate entry tunnel is able to filter substrates based on several properties, such as ligand size, geometry, and hydrophobicity (30). The R88C mutation reported here in strain SG 042 is predicted to affect susceptibility to azole drugs by affecting antifungal-ERG11 binding kinetics. One possible mechanism is through alteration of the protein surface and structural characteristics. The change of amino acid from arginine to cysteine represents a significant change in the surface properties at this position, as arginine is a positively charged and hydrophilic amino acid, whereas cysteine is uncharged and has the potential to form disulfide bonds. The R88C mutation also appears to open a secondary pore in the substrate entry tunnel (Fig. 3D), but its functional impact remains to be validated.

### The Y130F mutation is common in *M. arunalokei* and is likely to confer reduced azole susceptibility

A ERG11 Y130F mutation was detected in *M. restricta*, strain SG 040, and *M. arunalokei* strains SG 069 and SG 070 (Fig. 2; Table 2). This corresponds to higher MICs of 8 to 32 µg/mL for fluconazole (Table 2). This mutation has been identified in many other fungal species such as *Candida* and *Trypanosoma brucei* and *cruzi* (29). It was also detected in four *M. furfur* disease isolates (18) which have the phenotype of decreased fluconazole susceptibility. The substitution of tyrosine to phenylalanine affects the binding kinetics of fluconazole to ERG11 as the mutation occurs near a key binding residue (Fig. S2). The Y130F mutation results in the inability of ERG11 to form several hydrogen bonds with fluconazole, as noted in Fig. S2. Sagatova and colleagues found that in the *S. cerevisiae* ERG11 homolog, the equivalent residue (Y140) in the wild-type protein is able to form three hydrogen bonds with fluconazole and the heme group in ERG11, two of which are H_2_O-mediated (31). The inability to form these H-bonds in the Y140F mutant may result in weaker binding and lower affinity of fluconazole to the catalytic heme group in ERG11, resulting in reduced inhibitory effect of fluconazole. It is likely that a similar mechanism is conferring increased fluconazole resistance to the *M. arunalokei* Y130F mutant strains in this study.

### Reduced antifungal susceptibility is associated with increased ERG11 and ATM1 expressions and drug efflux pump activity

**Fig 4:**
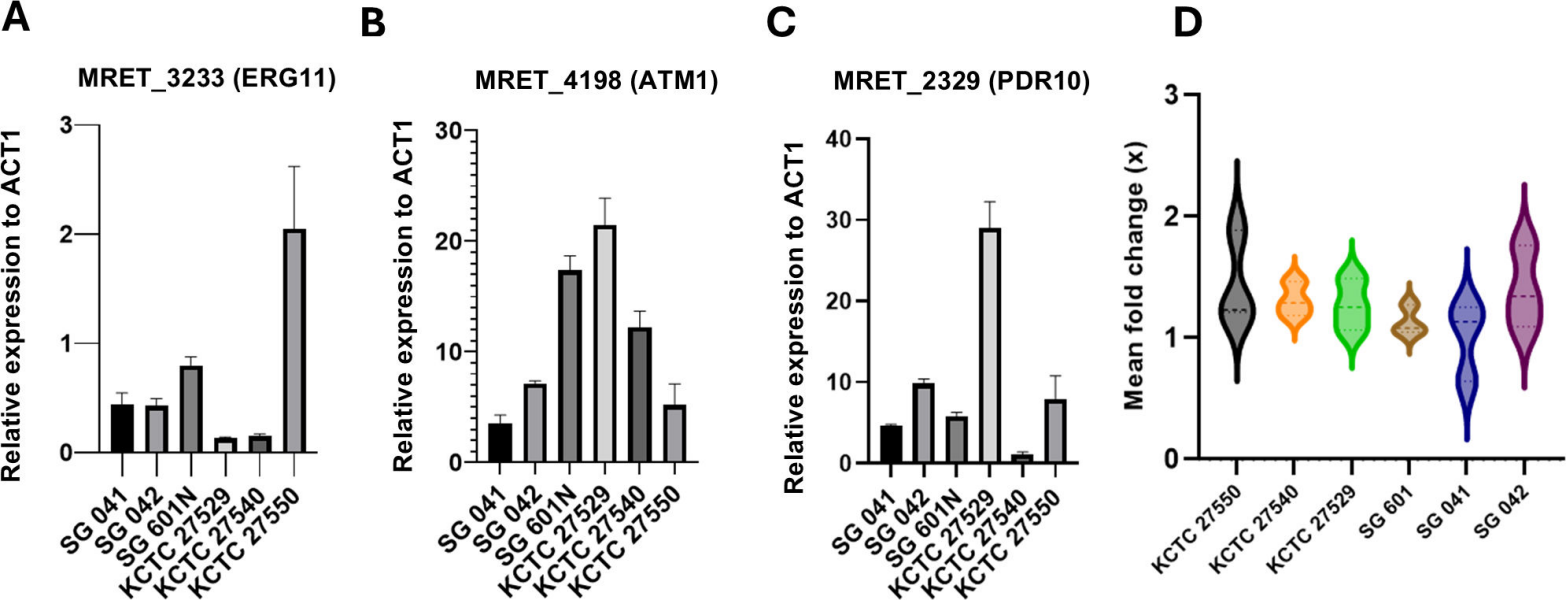
(A–C) Gene expression of ERG11, ATM1, and PDR10, respectively, as normalized to ACT1. Values shown represent mean ± SD. A one-way analysis of variance was used followed by Dunnett’s test (****P < 0.001) (Table S2). (**D**) Mean fold change of rhodamine 6G fluorescence signal at 30 min vs 0 min. Data are representative of three biological repeats.

In addition to ERG11 enzyme activity, drug efflux pumps such as PDR10 and ATM1 have also been identified to play a significant role in *Malassezia* antifungal susceptibility (5, 13, 14). To determine if increased gene expression is associated with reduced susceptibility, we performed gene expression analysis of the closest *S. cerevisi*ae homologs in *M. restricta* (Table 2) for ERG11, ATM1, and PDR10 using primers designed and validated for MRET_3233, MRET_4198, and MRET_2329, respectively. ERG11 and ATM1 expressions were highest in KCTC 27550 and KCTC 27529, respectively (Fig. 4A and B), consistent with previous reports that they are ketoconazole resistant. From the Singapore isolates, SG 601 had the highest ERG11 and ATM1 expression levels, which may account for its elevated fluconazole MIC values. SG 041 and SG 042 did not show significantly higher levels of ERG11, ATM1, or PDR10 expression compared to the other isolates. However, copy number quantitation suggests that their elevated MICs may be associated with increased ERG11, ATM1, and PDR10 copy numbers (Fig. S3).

Rhodamine 6G is a fluorescent dye which may be used to further evaluate yeast membrane efflux pump activity (32). *M. restricta* and *M. arunalokei* strains were loaded with rhodamine 6G and evaluated for dye efflux over 30 min. KCTC 27550, which was previously reported to be ketoconazole resistant, and SG 042, which was identified to harbor the novel ERG11 R88C mutation, were observed to have elevated drug efflux pump activity (Fig. 4D, ns, *P* > 0.05). This may contribute to their elevated MIC values (Fig. 1; Table 2).

## DISCUSSION

In this study, we evaluated the antifungal susceptibility profiles of 12 local isolates of *M. restricta* and *M. arunalokei* and analyzed their ERG11 DNA sequences and ERG11, ATM1, and PDR10 gene expressions to identify mechanisms that could account for their reduced antifungal susceptibility. Of the four non-synonymous ERG11 gene mutations observed in the local healthy isolates, one (R88C) from strain SG 042 was observed to be novel, and another (Y130F) from strains SG 040, 069, and 070 has been previously reported. The remaining two non-synonymous ERG11 gene mutations were observed to be associated with *M. arunalokei* species identification, although further genome sequencing would be required for validation. Due to the high degree of sequence similarity between *M. restricta* and *M. arunalokei* (3), and the latter being a relatively newly described species, there are limited verified reference genomes and strains available publicly for validation, and ITS sequencing is unable to reliably distinguish between them (33). It was also difficult to draw conclusions on *M. arunalokei* antifungal susceptibility as there have been no reports on the susceptibility of this species, and our work only covered five strains, four of which were locally derived.

Homology relates to proteins sharing a common ancestry, with conserved regions and structures. This may not imply a high percentage of sequence identity, and it has been demonstrated that, specifically for the P450 (CYP) protein family, homology modeling sees increased success when sequence identity is >20% even when the template is from another family (e.g., CYP1A2 in humans and CYP102 in bacteria) (34, 35). Based on the above, we would expect reasonable accuracy using our template-based *in silico* modeling approach for the same family of proteins (i.e., CYP51) with high sequence identity (e.g., ~47% for *M. restricta* ERG11 and *S. cerevisiae*) between two different species.

Based on our observations, we would anticipate that the R88C mutant would cause a net decrease in inhibitor availability to bind to the active site, resulting in a lower inhibitory effect of fluconazole on the catalytic activity of ERG11. To validate these observations, we attempted to generate the R88C mutant in other wild-type *M. restricta* and *M. furfur* strains but were not successful due to limitations in the *Agrobacterium tumefaciens*-mediated transformation approach, which is presently the only way to transform *Malassezia*. Future work may warrant the use of more powerful *in silico* models for quantitative measurement of ligand binding kinetics in these models, although the reliability of these approaches remains to be evaluated (36).

However, it is noted that isolated mutations in ERG11 may not be sufficient to induce changes in antifungal susceptibility (37). Multiple azole resistance mechanisms have been reported to be simultaneously and actively driving a resistant phenotype (38, 39). This was observed to be the case in *M. furfur,* where the generation of a Y130F mutant was not sufficient to change in antifungal susceptibility (13). The Y130F mutation was also observed to be common (two of five) in *M. arunalokei,* but the strains did not show any reduction in antifungal susceptibility. Thus, it is possible that the elevated MICs in SG 042 could be a combination of the ERG11 R88C mutation and an increased ATM1 gene copy number.

Changes in drug efflux pump activity and expression are the most prevalent cause of antifungal resistance and enable fungi to adapt rapidly to antifungal challenge (40). Strain SG 041 was not found to have ERG11 mutations but had elevated MICs to all antifungals tested apart from ketoconazole. However, it also did not have significantly higher expression of PDR10, ERG11, and ATM1 genes. Copy number quantitation (Fig. S3) suggests that the elevated MICs in SG 041 and SG 042 are likely to be mediated by an increase in ERG11, ATM1, and PDR10 copy numbers. It is likely that there are other key drug efflux pumps and enzymes which may be driving reduced antifungal susceptibility to selected azoles, and these are differentially expressed across all strains. Identifying key transcription factors in *Malassezia* upstream of these genes would be important in defining the molecular switch required to control emerging antifungal resistance.

Our results indicate that antifungal susceptibility is determined by a combination of factors including mutations, efflux pump activity, gene expression, and copy number. The background of the strain (e.g., healthy/diseased skin, prior exposure to antifungals) and the types of over-the-counter antifungal creams/shampoos available in the region are likely to drive/influence susceptibility patterns (41). The healthy skin microbiome serves as a reference for the emergence of new mutations and strain phenotypes which may give rise to opportunistic infection (42).

## SUPPLEMENTAL MATERIAL

Supplemental figures spectrum.00007-25-s0001.docx Fig. S1 to S3.

Table S1 spectrum.00007-25-s0002.xlsx List of primers used in this study.

Table S2 spectrum.00007-25-s0003.xlsx One-way ANOVA for MRET_3233, MRET 4198 and MRET 2329 gene expression.

Table S3 spectrum.00007-25-s0004.xlsx One-way ANOVA for MRET_3233, MRET 4198 and MRET 2329 copy number.

ASM does not own the copyrights to Supplemental Material that may be linked to, or accessed through, an article. The authors have granted ASM a non-exclusive, world-wide license to publish the Supplemental Material files. Please contact the corresponding author directly for reuse.
