# Supplementary material for: Non-synonymous ERG11 mutations in M. restricta and M. arunalokei: impact on azole susceptibility
Source: Microbiol Spectr. 2025 May 15;13(6):e00007-25. doi: 10.1128/spectrum.00007-25 (PMC12131850; doi:10.1128/spectrum.00007-25)
Supplement: Supplemental figures — Fig. S1 to S3. [file spectrum.00007-25-s0001.docx]

**Supplementary Material**

**Non-synonymous ERG111 mutations in *M. restricta* and *M. arunalokei*: impact on azole susceptibility**

*Cheryl Leong^1#^, Wisely Chua^1^, Chong Cheng-Shoong^2^, Lee Shi Mun^1^, Sebastian Maurer-Stroh^2,3^, Jung Won Hee^4^, Thomas L. Dawson, Jr^1,5#^*

^1^ A*STAR Skin Research Labs (A*SRL), Agency for Science, Technology and Research (A*STAR) & Skin Research Institute of Singapore (SRIS), 11 Mandalay Rd, #17-01, Singapore 308232, Republic of Singapore

^2^Bioinformatics Institute, Agency for Science, Technology and Research (A*STAR), Singapore, Singapore**,**

^3^Department of Biological Sciences and Yong Loo Lin School of Medicine, National University of Singapore (NUS), Singapore,^4^Chung-Ang University,

^5^Center for Cell Death, Injury & Regeneration, Departments of Drug Discovery & Biomedical Sciences and Biochemistry & Molecular Biology, Medical University of South Carolina, Charleston, SC

#Address correspondence to:

Cheryl Leong or Thomas Dawson

A*STAR Skin Research Labs

11 Mandalay Road, #17-01, Singapore 308232

Singapore

**Key words: antifungal, resistance, azoles, *Malassezia, ERG11***

**Supplementary Figures**

Supplementary Figure 1. Multiple Sequence alignment & Phylogenetic tree of ITS regions for *M. restricta* and *M. arunalokei*

Supplementary Figure 2. Model of the CYP51 Y130F mutant strain

Supplementary Figure 3. Mean normalized copy number of PDR10, CYP51 and ATM1 genes

**Supplementary Tables (Excel sheets)**

Supplementary Table 1. List of primers used in this study

Supplementary Table 2. One-way ANOVA for MRET_3233, MRET 4198 and MRET 2329 gene expression

Supplementary Table 3. One-way ANOVA for MRET_3233, MRET 4198 and MRET 2329 copy number

**Supplementary Figure 1.
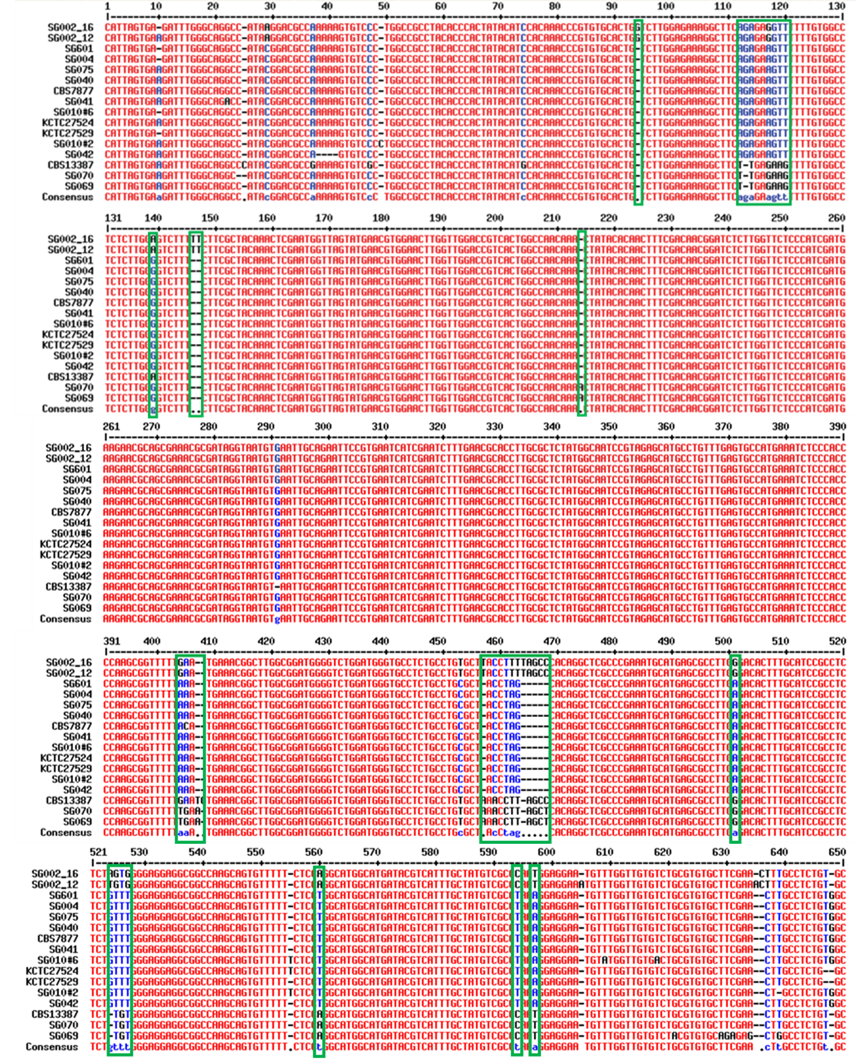
**

**A**

**
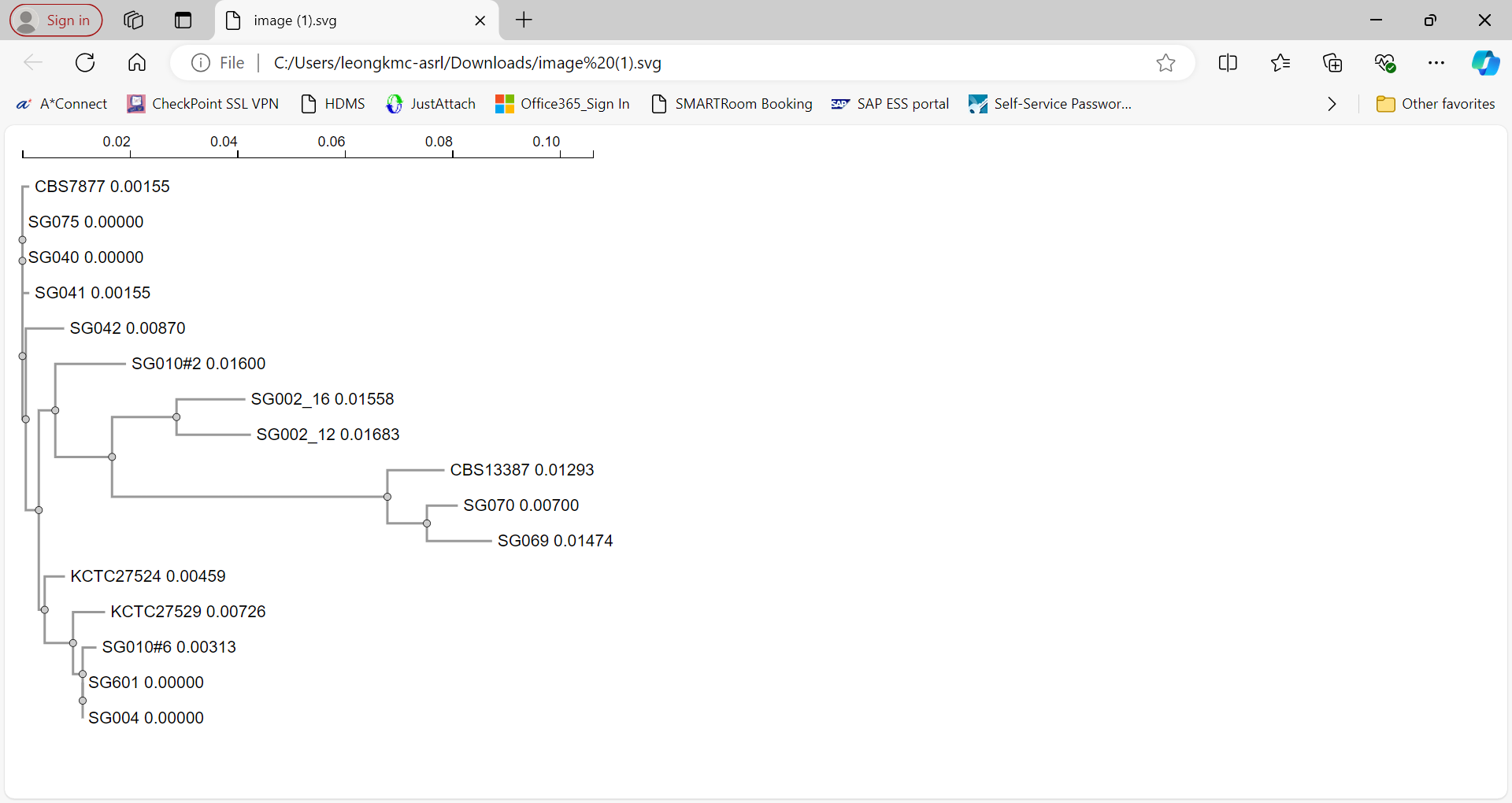
**

**B**

**Supplementary Figure 1**. (A) Multiple sequence alignment of the ITS regions of Singapore *Malassezia* isolates and the corresponding *M. restricta* and *M. arunalokei* reference strains. Boxes highlighted in green indicate regions of dissimilarity that are divergent from *M. restricta* ITS sequences and show more similarity to *M. arunalokei* and (B) Phylogenetic tree of ITS sequences indicates that likely *M. arunalokei* strains cluster separately from *M. restricta*.

**Supplementary Figure 2.**

**Supplementary Figure 2**. Model of the CYP51 Y130F mutant strain. The mutant F130 group (cyan) is superimposed upon the wildtype Y130 (red). Other residues within 7Å of fluconazole are shown in green - A112, Y116, M380 are hidden for clarity. Black dashed lines represent hydrogen bonds, which are formed between the wildtype and fluconazole, but not Y130F mutant and fluconazole. Image prepared with CHIMERA[22].

**Supplementary Figure 3.**

**Supplementary Figure 3**: Mean copy number of ERG11, ATM1, PDR10 genes respectively as normalized to ACT1. Values shown represent mean ± SD. A one-way ANOVA was used followed by Tukey’s test (****p<0.001),
